# Lack of *HXK2* Induces Localization of Active Ras in Mitochondria and Triggers Apoptosis in the Yeast *Saccharomyces cerevisiae*

**DOI:** 10.1155/2013/678473

**Published:** 2013-09-05

**Authors:** Loredana Amigoni, Enzo Martegani, Sonia Colombo

**Affiliations:** ^1^Department of Biotechnology and Biosciences, University of Milano-Bicocca, Piazza Della Scienza 2, 20126 Milan, Italy; ^2^SysBio Centre of Systems Biology, Piazza Della Scienza 2, 20126 Milan, Italy

## Abstract

We recently showed that activated Ras proteins are localized to the plasma membrane and in the nucleus in wild-type cells growing exponentially on glucose, while in the *hxk2*Δ strain they accumulated mainly in mitochondria. An aberrant accumulation of activated Ras in these organelles was previously reported and correlated to mitochondrial dysfunction, accumulation of ROS, and cell death. Here we show that addition of acetic acid to wild-type cells results in a rapid recruitment of Ras-GTP from the nucleus and the plasma membrane to the mitochondria, providing a further proof that Ras proteins might be involved in programmed cell death. Moreover, we show that Hxk2 protects against apoptosis in *S. cerevisiae*. In particular, cells lacking *HXK2 * and showing a constitutive accumulation of activated Ras at the mitochondria are more sensitive to acetic-acid-induced programmed cell death compared to the wild type strain. Indeed, deletion of *HXK2* causes an increase of apoptotic cells with several morphological and biochemical changes that are typical of apoptosis, including DNA fragmentation, externalization of phosphatidylserine, and ROS production. Finally, our results suggest that apoptosis induced by lack of Hxk2 may not require the activation of Yca1, the metacaspase homologue identified in yeast.

## 1. Introduction

In *Saccharomyces cerevisiae* the highly homologous genes *RAS1 *and *RAS2* encode small G-proteins that are activated by the guanine nucleotide exchange factors (GEFs), Cdc25 and Sdc25 [1, 2] and inactivated by the GTPase-activating proteins (GAPs), Ira1 and Ira2 [3]. GEFs and GAPs control the switch of the two small monomeric proteins between the active GTP-bound and the inactive GDP-bound state. The Ras proteins and the GPCR system [4–6] constitute two branches that modulate the activity of adenylate cyclase (Cyr1), according to the glucose availability in the environment. In turn Cyr1 [7] activates cAMP-dependent protein kinase (PKA) through cAMP. The amount of this second messenger is also regulated at the level of degradation by the two phosphodiesterases, Pde1 and Pde2. PKA plays a major role in the modulation of metabolism, stress resistance, cell growth, proliferation, morphogenesis, and aging [8]. 

Recently, our group expressed a probe consisting of a GFP fusion with a trimeric Ras Binding Domain of Raf1 (eGFP-RBD3), which binds Ras-GTP with a much higher affinity than Ras-GDP, to investigate the localization of active Ras in wild-type and in mutant strains in the cAMP/PKA pathway [9]. Our results showed that in W303-1A wild-type cells the probe is localized essentially at the plasma membrane and in the nucleus, while in *hxk2*Δ cells the fluorescent signal accumulated in internal membranes and mitochondria [9]. This peculiar localization of activated Ras2 was previously found in *S. cerevisiae* cells lacking Whi2p function, a protein known to influence cell cycle exit under conditions of nutritional stress [10]. The loss of Whi2p function led to accumulation of damaging ROS and cell death that displayed the hallmarks of apoptosis. More recently, it has been shown that also in mammalian cells, translocation of activated K-RAS protein to mitochondria caused mitochondrial dysfunction and increased ROS generation [11].

Apoptosis plays a crucial role in embryogenesis, development, tissue homeostasis, and disease control in multicellular organisms. In the last two decades the budding yeast *S. cerevisiae* has become a useful model organism for studying this process [12–15]. The basic molecular machinery executing programmed cell death is phylogenetically conserved in yeast as well as animals. Yeast orthologues of mammalian genes related to apoptosis coding for caspase (Yca1), the apoptosis-inducing factor (Aif1), the AIF-homologous mitochondrion-associated inducer of death (Ndi1), the serine protease OMI (Nma111), the endonuclease G (Nuc1), and the endo-/exonuclease Tat-D (scTat-D) [12, 15–20] have been characterized. The apoptotic pathway in *S. cerevisiae* can be activated by several mutations, including *cdc48-S565G* [13], the inactivation of the UBP10 gene coding for a deubiquitinating enzyme [21] or by overexpression of the mammalian apoptotic cell death regulator Bax [22]. Moreover apoptotic cell death is also induced by exogenous toxic agents such as hydrogen peroxide [23], formic acid [24], acetic acid [25], and others. In particular, acetic-acid-induced apoptosis has been investigated in detail, and it has been shown that ROS accumulation and release of cytochrome c to the cytosol take place and that H_2_O_2_ is a trigger for acetic-acid-induced apoptosis [26–29]. In addition, at least two death pathways can be activated in yeast acetic-acid-induced apoptosis, one is dependent on cyt c release, which requires *YCA1* and the other(s) is independent of it [16, 30]. The yeast caspase Yca1 can protect yeast cells against multiple distinct forms of lethal insults, such as exposure to metals (iron, manganese, cadmium), to low doses of valpronic acid and the previous mentioned acetic acid, to toxins produced by virus killer toxins and others [31]. On the other side, in many instances, Yca1 is not necessary for cell death. For example, external stimuli such as formic acid or copper, or apoptosis derived from defective N-glycosylation in cells lacking Ost2p, the yeast homolog of the mammalian defender of apoptosis-1, are independent of *YCA1* [31].

In this work we provide data indicating that a correlation exists between programmed cell death and localization of active Ras proteins to mitochondria. First of all, we show that addition of acetic acid to wild-type cells causes within five minutes a delocalization of the eGFP-RBD3 probe from plasma membrane and nucleus to mitochondria. Furthermore, we show that in *hxk2*Δ cells, showing a constitutive localization of active Ras at the mitochondria, addition of acetic acid causes an increase of ROS accumulation, mitochondrial dysfunction, and cell death compared with the wild-type strain. It is known that hexokinase 2 functions as a glycolytic enzyme in the cytoplasm and as a regulator of gene transcription of several Mig1-regulated genes in the nucleus [32, 33]. In this paper, we provide data showing a new role for hexokinases 2 as an antiapoptotic factor in this microorganism.

## 2. Materials and Methods

### 2.1. Yeast Strains and Media

Strains used in this study: W303-1A (*MATa ade2-1 can1-100 his3-11,15 leu2-3112 trp1-1 ura3-1*) [34]; YSH310 (*MATa* W303-1A with *hxk2::LEU2*) [34]; *yca1*Δ (*MATa* W303-1A with *yca1::URA3*) (this study); *hxk2*Δ *yca1*Δ (YSH310 with *yca1::URA3*) (this study); W303-1A [peGFP-RBD3] [9]; YSH310 [YCp*RAS2* ^Val19^], are obtained by transforming YSH310 with plasmid YCp*RAS2* ^Val19^ [35]. The *yca1*Δ and the* hxk2*Δ *yca1*Δ strains were generated by one-step gene disruption [36] from the wild-type W303-1A and the YSH310 strains, respectively, using specific primers previously described [37] and kindly provided by M. Vai, University of Milano-Bicocca. 

Synthetic complete media (SD) contained 2% glucose, 6.7 g/L YNB w/o aminoacids (Becton and Dickinson Italia, Buccinasco) and the proper selective drop-out CSM (Complete Synthetic Medium, supplied by BIO101, California, USA). Culture density was measured with a Coulter Counter (Coulter mod. Z2) on mildly sonicated, diluted samples. YEPD plates contained 2% w/v glucose, 2% w/v peptone, 1% yeast extract, and 2% agar. 

### 2.2. Acetic Acid Treatment

Cells were grown at 30°C to exponential phase (1-2 × 10^7^ cells/mL) in SD medium, harvested, resuspended (10^7^ cells/mL) in fresh SD medium adjusted to pH 3.0 (set with HCl), and treated with acetic acid (Riedel-deHaen) at the indicated concentration (between 0 and 120 mM). Cells were incubated for up to 200 minutes at 30°C with shaking (160 rpm).

### 2.3. Fluorescence Microscopy to Detect Active Ras Localization

W303-1A cells were grown in SD medium at 30°C until exponential phase and treated with 40 mM acetic acid as described previously. Both treated and untreated cells were incubated with the mitochondrial marker Rhodamine B hexyl ester perchlorate (Molecular Probes, Eugene, OR, USA) 100 nM final concentration for about 5 min before imaging. Subsequently, 40 *μ*L of cells suspension was seeded on concanavalin A (Sigma-Aldrich, Milano, Italy) coated cover glass for 10 min (100 *μ*g/mL). The cover glass was washed 4 times using the proper medium and put on top of a Thoma chamber. Images were acquired with a Nikon Eclipse E600 microscope equipped with a 60X, 1.4 oil Plan-Apochromat objective and a standard FITC filter set for GFP fluorescence. Images were recorded digitally using a Leica DC 350F camera and processed using Adobe Photoshop (Adobe Systems, Inc.).

### 2.4. Acetic Acid Sensitivity

This assay was performed essentially as described by Casatta et al. [38]. Exponential-phase cells were harvested and resuspended (10^7^ cells/mL) in SD medium adjusted either at pH 5.3 or at pH 3.0 (set with HCl) and containing 0, 40, 80, or 120 mM acetic acid. Cells were incubated for 200 min at 30°C with shaking (160 rpm). After treatment, cells (10-fold serial dilutions) were spotted onto YEPD plates and incubated at 30°C for 3 days.

### 2.5. Viability Assay

Cells were grown in SD medium at 30°C until exponential phase and treated with acetic acid as described previously. At different times (0, 30, 60, and 200 minutes) during acetic acid treatment, cell number was calculated and 400 cells were plated. Viability was determined by measuring colony-forming units (cfu) after 2 days of growth on YEPD agar plates at 30°C. The percentage of viable cells resulted in dividing the number of surviving colonies of the treated sample by the number of surviving colonies of the same culture before acid acetic addition. 

### 2.6. Dihydrorhodamine 123 (DHR123) Staining

ROS (reactive oxygen species) were detected with DHR123 (Sigma Aldrich) essentially as described by Madeo et al. [23]. Cells were grown in SD medium at 30°C until exponential phase and treated with acetic acid as described previously. DHR123 was added directly to the culture medium at the final concentration of 5 *μ*g/mL from a 2.5 *μ*g/*μ*L stock solution. After 2 hours of incubation, cells were diluted to 10^6^ cells/mL and analyzed using a FACScan instrument (Becton Dickinson) at low flow rate with excitation and emission settings of 488 and 525–550 nm (filter FL 1). A total of 20.000 events were acquired for each sample and data were processed using WinMDI 2.9 software.

### 2.7. 4′,6-Diamidino-2-phenylindole (DAPI) Staining

Cells were fixed with 3.7% formaldehyde for 30 minutes, stained with 2 *μ*g/mL of DAPI for 10 minutes, washed with distilled water, and resuspended in 50% glycerol solution. Images were acquired with a Nikon Eclipse E600 fluorescence microscope using a DAPI filter, recorded digitally using a Leica DC 350F camera, and processed using Adobe Photoshop (Adobe Systems, Inc.).

### 2.8. Annexin V and Propidium Iodide (PI) Staining

(FITC) conjugated recombinant Annexin V (Immuno Tools) was used for the detection of phosphatidylserine exposed in the membrane of apoptotic cells. Cells were harvested after 200 minutes of acetic acid treatment, as reported previously, washed with sorbitol buffer (1 M sorbitol, 0.1 M NaH_2_PO_4_, pH 8.0), and the cell wall was digested with Zymolyase 20T (Seikagaku Biobusiness Corporation) for about 35 minutes at 37°C. Cells were then washed two times with binding buffer (10 mM Hepes/NaOH pH 7.4, 140 mM NaCl, 2.5 mM CaCl_2_, 1.2 M sorbitol). Spheroplasts were resuspended in 35 *μ*L of binding buffer and incubated with 2.5 *μ*L of Annexin V and 2 *μ*L of a PI (Fluka) working solution (50 *μ*g/mL) for 15 minutes in the dark at room temperature. After staining, the samples were resuspended in binding buffer and analyzed using a FACScan instrument (Becton Dickinson) using FL 1-H filter on *x*-axis and FL 2-H filter on *y*-axis. A total of 30.000 events were acquired for each sample, and data were processed using WinMDI 2.9 software. 

### 2.9. 3,3′-Dihexyloxacarbocyanine Iodide (DiOC6) Staining

The mitochondrial morphology and membrane potential were assessed by staining with DiOC_6_ (Molecular Probes, Invitrogen). Cells were grown in SD medium at 30°C until exponential phase and treated with acetic acid as described previously. DiOC6 was added directly to the culture medium at the final concentration of 175 nM for 15 minutes in the dark at room temperature. After staining, the cells were analyzed using a FACScan instrument (Becton Dickinson) using FL1-H filter. A total of 20.000 events were acquired for each sample, and data were processed using WinMDI 2.9 software. Images were also acquired with a Nikon Eclipse E600 microscope equipped with a 60X, 1.4 oil Plan-Apochromat objective, and a standard FITC filter set. 

## 3. Results and Discussion

### 3.1. Effect of Acetic Acid on the Localization of Active Ras in Glucose-Growing Cells

In a recent study we showed that active Ras proteins accumulated mainly in the plasma membrane and in the nucleus in exponentially growing wild-type cells, while they accumulated in mitochondria in cells deleted in the *HXK2* gene, indicating that this enzyme was involved in the proper localization of these small GTPases [9]. The role played by active Ras in mitochondria is not known, although other reports have found Ras-GTP associated with these organelles, both in mammals and lower eukaryotes, and have linked it to cancer and apoptosis. Leadsham et al. [10] demonstrated that in *S. cerevisiae* loss of Whi2p function, a protein known to influence cell cycle exit under conditions of nutritional depletion determined an aberrant accumulation of activated Ras at the mitochondria. In this mutant, the failure to shut down Ras signalling by addressing Ras to the vacuole would lead to mitochondrial dysfunction, accumulation of damaging ROS, and cell death. More recently, Hu et al. [11], using a tetracycline inducible model, demonstrated that in mammalian cells association of K-ras^G12V^ proteins with mitochondria induced mitochondrial dysfunction, increased ROS accumulation, and a metabolic switch from oxidative phosphorylation to glycolysis.

**Figure 1:**
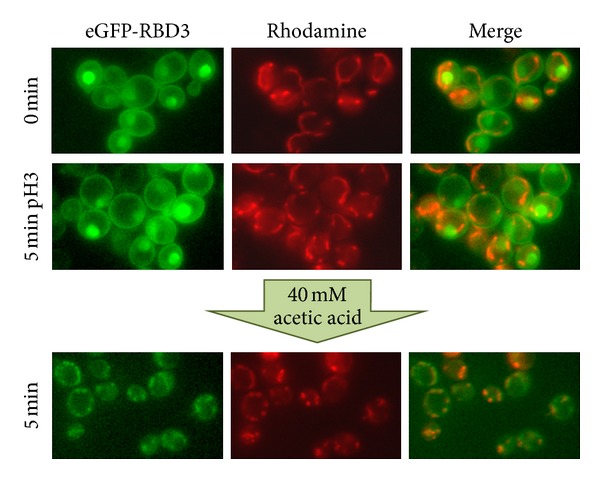
Localization of active Ras in the W303-1A wild-type strain after addition of 40 mM acetic acid. Cells transformed with YEpeGFP-RBD3 were grown in 2% glucose medium at 30°C until exponential phase, collected by centrifugation, and resuspended in medium adjusted to pH 3.0. Cells were then photographed with a Nikon fluorescence microscope, before and after addition of 40 Mm acetic acid. Colocalization of eGFP fluorescence and the red-fluorescent Rhodamine B hexyl ester is clearly visible five minutes after addition of the apoptotic stimulus.

To investigate whether a correlation exists between the mitochondrial localization of active Ras and programmed cell death, we evaluated the effect of the addition of acetic acid, a well-known apoptotic stimulus [25], to wild-type cells expressing the eGFP-RBD3 probe on the localization of Ras-GTP proteins. To this aim, exponentially growing cells were collected by centrifugation, resuspended in low pH medium (pH 3.0), and pictures were taken at the fluorescence microscope, before and after addition of 40 mM acetic acid. Our results showed that within 5 minutes this apoptotic stimulus caused the localization of active Ras proteins exclusively to the mitochondria, reinforcing the hypothesis of the involvement of these proteins in programmed cell death (Figure 1). In wild-type cells resuspended in low pH medium for 5 minutes without acetic acid added, active Ras proteins accumulated mainly in the plasma membrane and in the nucleus, indicating that acidification of the medium did not influence the localization of these small G proteins (Figure 1). A mitochondrial localization of Ras-GTP was also observed when a low concentration of H_2_O_2_ was added to the medium (data not shown), indicating that delocalization of active Ras to these organelles is a more general response to different apoptotic stimuli and suggesting that mitochondrial localization of Ras2-GTP is actually important for the induction of apoptosis in yeast.

### 3.2. Deletion of HXK2 Enhances Cell Death and Increases Intracellular ROS Levels

**Figure 2:**
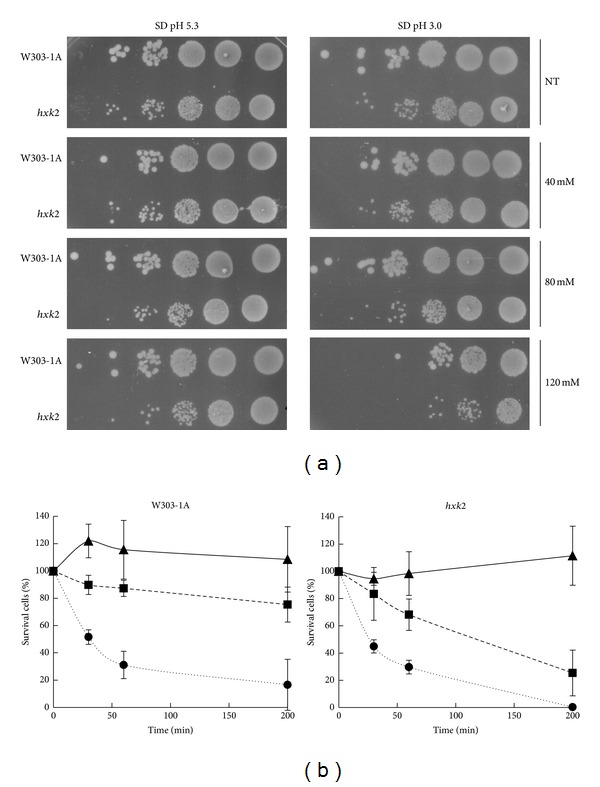
*hxk2*Δ cells exhibit inhibition of cell growth and hypersensitivity to acetic acid. (a) W303-1A and *hxk2*Δ cells were harvested and resuspended (1-2 × 10^7^ cells/mL) in SD medium adjusted either at pH 5.3 or at pH 3.0 (set with HCl) in the absence (NT) or in the presence of 40, 80, or 120 mM acetic acid. Cells were incubated for 200 min at 30°C with shaking (160 rpm). After treatment, cells were harvested and resuspended at the same concentration (10^8^ cells/mL) in milliQ water. 5 microliter from a concentrated suspension and from 10-fold dilutions of each culture was spotted onto YEPD plates and incubated at 30°C for 3 days. (b) Cell survival of W303-1A and *hxk2*Δ strains. Cell viability of W303-1A and *hxk2*Δ untreated cells (▲) or treated with 80 mM (■) and 120 mM (*⚫*) acetic acid was analyzed at indicated times by measuring colony-forming units (cfu) after 2 days of growth at 30°C. Cell survival (100%) corresponds to the cfu at time zero. The means of 4 independent experiments with standard deviations are reported.

The results presented previously suggest that localization of active Ras proteins to mitochondria might be involved in programmed cell death, since these proteins are localized to mitochondria following addition of acetic acid. Since in glucose-growing *hxk2*Δ cells, active Ras is constitutively located in mitochondria [9], we next analyzed the behavior of the *hxk2*Δ mutant under conditions that caused apoptosis. While the growth of *hxk2*Δ mutant was almost indistinguishable from the wild-type strain at pH 5.3, it exhibited an increased sensitivity to acetic acid stress at low pH (Figure 2(a)), suggesting that Hxk2 is required for normal tolerance to acetic acid treatment. Cell survival of wild-type and *hxk2*Δ cells after induction of apoptosis with acetic acid was further tested in a plating assay. After treatment with different concentrations (80 and 120 mM) of acetic acid for up to 200 minutes at 30°C, *hxk2*Δ cells showed a significant dose-dependent reduction in cell survival when compared with wild-type cells (Figure 2(b)). Taken together these data suggest that Hxk2 is required for normal tolerance to acetic acid treatment.

**Figure 3:**
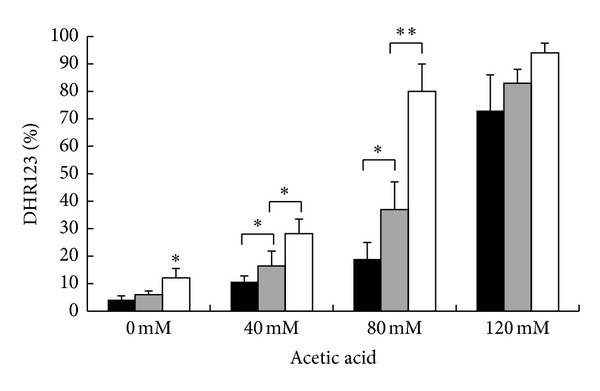
ROS accumulation in W303-1A, *hxk2*Δ, and *hxk2*Δ cells expressing the activated *Ras2* ^Val19^ allele after treatment with acetic acid. W303-1A (black bars), *hxk2*Δ (gray bars), and *hxk2*Δ cells expressing the activated Ras2^Val19^ allele (white bars) exponentially growing cells were treated with different concentrations (40–80–120 mM) of acetic acid for 200 minutes at 30°C. ROS accumulation was assayed using the dye dihydrorhodamine 123 (DHR123) by flow cytometry. The means of 3 independent experiments with standard deviations are reported. Student's *t*-test *P < 0.05 and **P < 0.01.

Since an aberrant accumulation of activated Ras in mitochondria accompanied to accumulation of ROS has already been reported both in mammals and yeast [10, 11] and since ROS play a pivotal role in apoptotic cell death, FACS analyses were performed to evaluate the accumulation of ROS in wild-type and *hxk2*Δ cells treated with different concentrations of acetic acid for 200 min at 30°C. In particular, DHR123 was used to determine the accumulation of ROS in the cells, since this compound can easily permeate them and can be quantitatively oxidized to a green fluorescent product in the presence of ROS. The percentage of oxidized R123 was about the double for *hxk2*Δ cells compared to wild-type cells, 200 min after treatment with either 40 or 80 mM acetic acid (Figure 3). To substantiate the hypothesis that an aberrant accumulation of activated Ras in mitochondria might lead to mitochondrial dysfunction and accumulation of damaging ROS, we expressed the dominant active *RAS2* ^Val19^ allele, which was reported to show a much higher level of Ras2-GTP [39], in *hxk2*Δ cells. Our results showed that indeed the expression of this allele in the* hxk2*Δ background caused a further increase in the level of ROS, both in growing cells and after treatment with acetic acid (Figure 3). These data suggest that conditions that presumably cause a higher mitochondrial accumulation of active Ras may contribute to accumulation of ROS and cell death. 

### 3.3. Loss of Hxk2 Causes an Increase of Both Apoptosis and Necrosis

**Figure 4:**
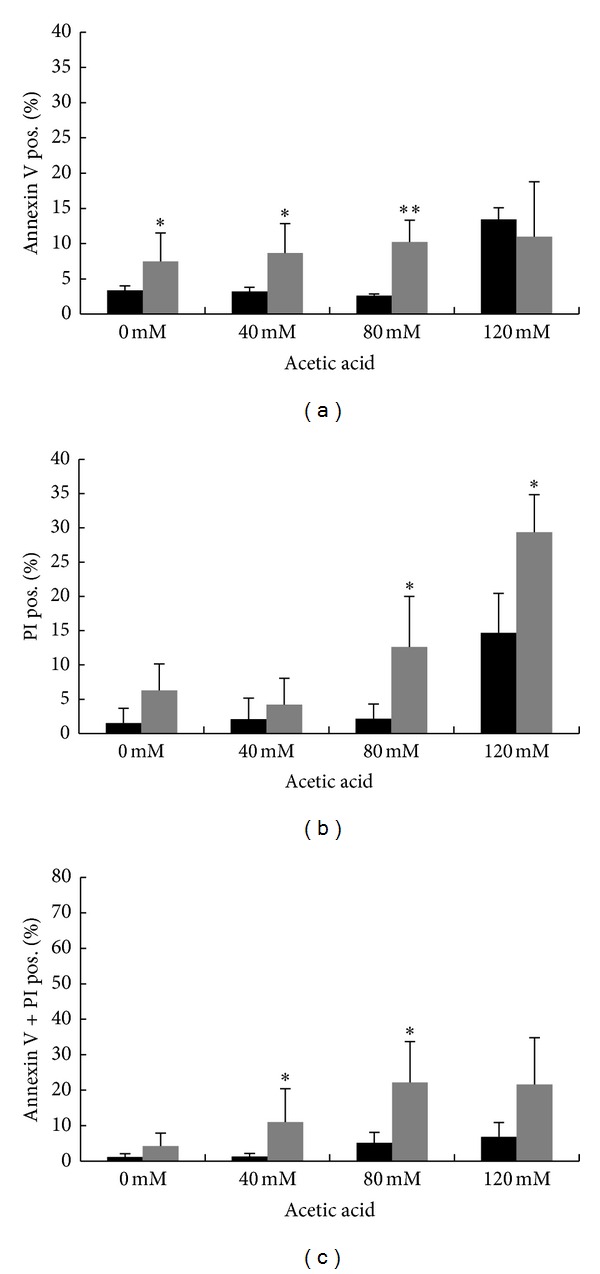
The occurrence of acetic-acid-induced cell death in *hxk2*Δ cells is characterized by markers of both apoptosis and necrosis. Assessment of cell death by FITC-coupled annexin V and PI staining. W303-1A (black bars) and *hxk2*Δ (gray bars) exponentially growing cells were treated with different concentrations (40–80–120 mM) of acetic acid for 200 minutes at 30°C, before being processed for determination of phosphatidylserine externalization and membrane integrity by flow cytometry. 30000 events have been evaluated. The means of 3 independent experiments with standard deviations are reported. Student's *t*-test *P < 0.05 and **P < 0.01.

The results presented previously suggest that Hxk2 might have an antiapoptotic activity. To better characterize the nature of cell death triggered by addition of acetic acid to *hxk2*Δ cells, we quantified phenotypic changes indicative of apoptosis. While apoptotic DNA condensation was detected by DAPI staining, combined Annexin V/propidium iodide (PI) staining was used to discriminate between early apoptotic (Annexin V+/PI−), late apoptotic/secondary necrotic (Annexin V+/PI+), and necrotic (Annexin V−/PI+) deaths. DAPI staining showed that nuclei of untreated cells were round, while the nuclear DNA was condensed in both wild-type and *hxk2*Δ cells treated with 80 mM acetic acid, being the extent of this phenotype more pronounced for the mutant strain compared to the wild-type strain (data not shown). Annexin V/propidium iodide (PI) staining revealed that *hxk2*Δ-facilitated cell death was accompanied by an increase in both apoptotic and necrotic markers (Figure 4). In particular, untreated *hxk2*Δ cells showed a higher percentage of both early apoptotic and necrotic cells compared with the wild-type strain, which further increased upon treatment with acetic acid. 

### 3.4. Deletion of HXK2 Causes Hyperpolarization of Mitochondria

**Figure 5:**
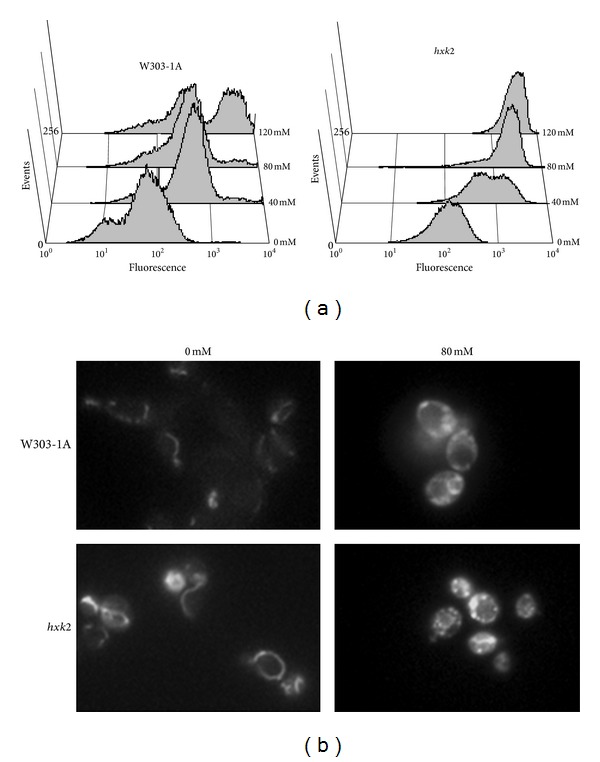
Mitochondrial membrane potential and morphology in W303-1A and *hxk2*Δ strains upon acetic acid treatment. W303-1A and *hxk2*Δ exponentially growing cells were treated with different concentrations (40–80–120 mM) of acetic acid for 200 minutes at 30°C. DiOC_6_ uptake was assessed using both flow cytometry (a) and fluorescence microscopy (b). Photomicrographs illustrate the alteration of the tubular mitochondrial network to clustered mitochondrial morphology, particularly exacerbated in *hxk2*Δ cells after acetic acid treatment.

Mitochondrial membrane potential (ΔΨ_*m*_) is a useful indicator of mitochondrial function. To measure ΔΨ_*m*_, we used the cationic lipophylic dye DiOC_6_, which accumulates in mitochondria in accordance with ΔΨ_*m*_. Wild-type and *hxk2*Δ cells were treated with different concentration of acetic acid (40–80–120 mM) for 200 min at 30°C, collected, and stained with this dye. FACS analysis showed that mitochondrial membrane potential clearly increased in both wild-type and *hxk2*Δ cells after treatment with acetic acid, compared with untreated control cells, with the hyperpolarization of mitochondria being much stronger in the mutant cells compared with wild-type cells (Figure 5(a)). In parallel, mitochondrial morphology was assessed by using DiOC_6_, both in wild-type and *hxk2*Δ cells. At low concentration, this dye specifically stains the mitochondrial membranes in a manner that depends on membrane potential and can be observed by fluorescence microscopy. Before treatment with acetic acid, both wild-type and *hxk2*Δ cells displayed a tubular mitochondrial morphology, indicating that these mitochondria were healthy and possessed a membrane potential (Figure 5(b)). By contrast, the mitochondrial membranes present in both wild-type and *hxk2*Δ cells appeared rounded and highly fragmented after treatment with acetic acid (Figure 5(b)). This conversion of mitochondrial morphology from tubular to punctuate structures is likely to occur by excessive mitochondrial fission and has already been observed in yeast apoptosis induced by acetic acid treatment [40]. Importantly, DiOC_6_ staining was greatly increased and more pronounced in *hxk2*Δ cells compared to wild-type cells after treatment with acetic acid, confirming the increase of fluorescence observed in these mutant cells by FACS analysis. These data indicate that both in wild-type and *hxk2*Δ cells, acetic acid treatment caused hyperpolarization of mitochondrial membrane with consequent damage of these organelles and loss of functionality. This effect was more pronounced in *hxk2*Δ cells compared to wild-type cells.

### 3.5. Acetic-Acid-Induced Cell Death in the *hxk2*Δ Strain Is Yca1 Independent

**Figure 6:**
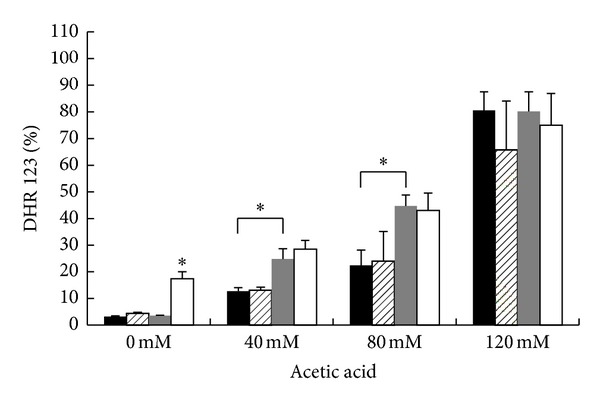
Effect of *YCA1* deletion on ROS accumulation after treatment with acetic acid. W303-1A (black bars), *yca1*Δ (black hatched bars), *hxk2*Δ (gray bars), and *hxk2*Δ*-yca1*Δ (white bars) exponentially growing cells were treated with different concentrations (40–80–120 mM) of acetic acid for 200 minutes at 30°C. ROS accumulation was assayed by flow cytometry using the dye dihydrorhodamine 123 (DHR123). Student's *t*-test *P < 0.05.

**Figure 7:**
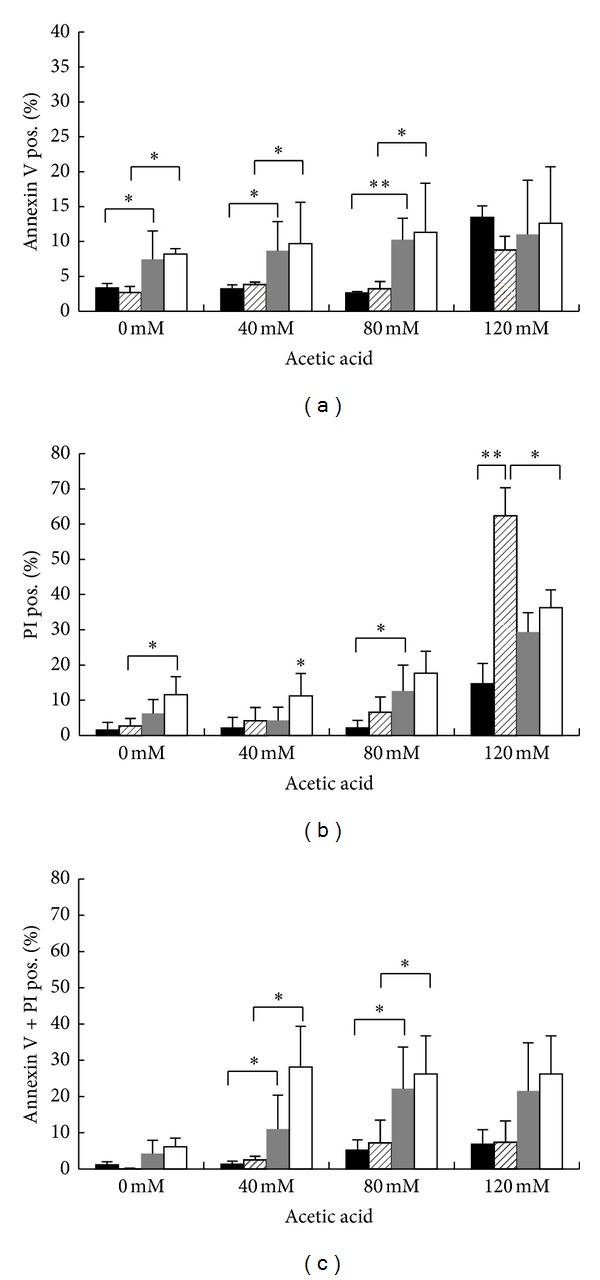
Effect of *YCA1* deletion on cell death after treatment with acetic acid. W303-1A (black bars), *yca1*Δ (black hatched bars), *hxk2*Δ (gray bars) and *hxk2*Δ*-yca1*Δ (white bars) exponentially growing cells were treated with different concentrations (40–80–120 mM) of acetic acid for 200 minutes at 30°C. Cell death was assessed by flow cytometry using FITC-coupled annexin V and PI co-staining to determinate the externalization of phosphatidylserine and the membrane integrity. The means of 3 independent experiments with standard deviations are reported. Student's *t*-test *P < 0.05 and **P < 0.01.

At least two death pathways can be activated in yeast acetic-acid-induced apoptosis, one dependent on cytochromec release, which requires *YCA1*, and the other(s) independent of it [16, 30]. Consequently, we next investigated whether the yeast caspase Yca1 might be involved in acetic-acid-induced cell death in *hxk2*Δ cells. In particular, to assess whether Yca1 plays a role in acetic-acid-induced ROS accumulation in *hxk2*Δ cells, we treated *hxk2*Δ and *hxk2*Δ *yca1*Δ cells with different concentration of acetic acid (40–80–120 mM) for 200 min at 30°C and measured the ROS accumulation. Our results showed that deletion of *YCA1 *in* hxk2*Δ cells had no effect on acetic-acid-induced ROS accumulation (Figure 6), suggesting that the generation of ROS in *hxk2*Δ cells upon acetic acid stress is Yca1 independent. However, the level of intracellular ROS was consistently higher in the* hxk2*Δ *yca1*Δ double mutant growing on glucose medium compared with the *hxk2*Δ mutant. Similarly, Du et al. [24] showed that formic acid induced Yca1-independent apoptosis-like cell death and that the burst of ROS during cell death occurred earlier and at a higher level in the *yca1*Δ strain than in the wild-type strain. Moreover, Khan et al. [41] determined the level of oxidized proteins in yeast cells under H_2_O_2_ stress and showed that lack of Yca1 abrogated apoptosis but elevated intracellular oxidized proteins compared with wild-type cells. Consequently, both our results and data in the literature [24, 41] clearly suggest a linkage between ROS production and Yca1 activation during apoptosis in yeast. Finally, cytofluorometric quantification of phosphatidylserine externalization and/or membrane permeabilization (annexin V/PI costaining) further confirmed that Yca1p did not influence the acetic-acid-induced cell death in *hxk2*Δ cells (Figure 7). These results strongly suggest that apoptosis induced by acetic acid in *hxk2*Δ cells is largely Yca1 independent. 

## 4. Conclusion

Several studies indicate that the Ras protein may be physically associated with mitochondria, both in yeast and mammals [10, 11, 42–44]. This study provides results indicating that association of active Ras to these organelles might be linked to apoptosis. First of all, we show that addition of acetic acid, a well-known apoptotic stimulus in *S. cerevisiae*, to growing wild-type cells determined a delocalization of active Ras proteins from nuclei and plasma membrane to mitochondria. Furthermore, we show that addition of acetic acid to* hxk2*Δ cells, showing a constitutive localization of active Ras in mitochondria [9], enhanced ROS production and cell death compared with wild-type cells. Moreover, expression of the dominant active *RAS2* ^Val19^ allele in the *hxk2*Δ background caused an even higher increase in the level of ROS, both in growing cells and after treatment with acetic acid, providing a further proof to the hypothesis that conditions that presumably cause a higher mitochondrial accumulation of active Ras may contribute to accumulation of ROS and cell death. We also show that, in *hxk2*Δ cells, addition of acetic acid increased cell death compared with the wild-type strain with the typical markers of apoptosis, highlighting a new role for Hexokinase 2, as an antiapoptotic factor in *S. cerevisiae *cells. However at this stage we cannot exclude a correlation between the well-known functions of Hxk2 in glucose repression and signalling and the role of Ras in inducing apoptosis.

Finally, in this study we show that lack of Hxk2 induces apoptosis via a mitochondria-mediated pathway without metacaspase Yca1 involvement, since deletion of *YCA1* in the *hxk2*Δ background did not abrogate the acetic-acid-induced accumulation of ROS and did not decrease the percentage of both apoptotic and necrotic cells.
